# 3-Amino-2-methyl-4-oxo-3,4-dihydro­quinazolin-1-ium *p*-toluene­sulfonate monohydrate

**DOI:** 10.1107/S1600536809013841

**Published:** 2009-04-18

**Authors:** Mohammad Arfan, M. Nawaz Tahir, Rasool Khan, Mohammad S. Iqbal

**Affiliations:** a University of Peshawar, Institute of Chemical Sciences, Peshawar 25120, Pakistan; b University of Sargodha, Department of Physics, Sargodha, Pakistan; c Government College University, Department of Chemistry, Lahore, Pakistan

## Abstract

In the title hydrated mol­ecular salt, C_9_H_10_N_3_O^+^·C_7_H_7_O_3_S^−^·H_2_O, the cation is protonated at a quinazolinone N atom and an intra­molecular N—H⋯O hydrogen bond occurs. In the crystal structure, inter­molecular N—H⋯O and O—H⋯O hydrogen bonds and C—H⋯O, C—H⋯π and weak aromatic π–π stacking inter­actions [centroid–centroid separations = 3.8648 (12) and 3.9306 (13) Å] help to establish the packing; a short S=O⋯π contact is also seen.

## Related literature

For a related structure, see: Atkinson & Meades (2000 ▶). For background on the properties of cyclic amidines and quinazolinones, see: Glaser & Traber (1984 ▶); Havera (1979 ▶); Hori *et al.* (1990 ▶); Liverton *et al.* (1998 ▶). For graph-set notation, see: Bernstein *et al.* (1995 ▶).
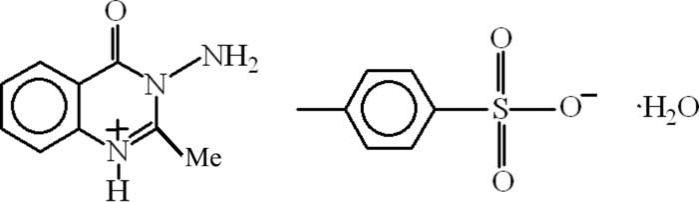


## Experimental

### 

#### Crystal data

C_9_H_10_N_3_O^+^·C_7_H_7_O_3_S^−^·H_2_O
*M* *_r_* = 365.40
Monoclinic, 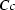

*a* = 20.838 (1) Å
*b* = 6.2769 (3) Å
*c* = 14.7897 (7) Å
β = 116.676 (1)°
*V* = 1728.56 (14) Å^3^
*Z* = 4
Mo *K*α radiation
μ = 0.22 mm^−1^
*T* = 296 K
0.28 × 0.24 × 0.20 mm

#### Data collection

Bruker Kappa APEXII CCD diffractometer
Absorption correction: multi-scan (*SADABS*; Bruker, 2005 ▶) *T*_min_ = 0.935, *T*_max_ = 0.958
9546 measured reflections
4390 independent reflections
3952 reflections with *I* > 2σ(*I*)
*R*_int_ = 0.022

#### Refinement

*R*[*F* ^2^ > 2σ(*F* ^2^)] = 0.034
*wR*(*F* ^2^) = 0.091
*S* = 1.00
4390 reflections
243 parameters
2 restraints
H atoms treated by a mixture of independent and constrained refinement
Δρ_max_ = 0.19 e Å^−3^
Δρ_min_ = −0.22 e Å^−3^
Absolute structure: Flack (1983 ▶), 2109 Friedal pairs
Flack parameter: 0.04 (5)

### 

Data collection: *APEX2* (Bruker, 2007 ▶); cell refinement: *SAINT* (Bruker, 2007 ▶); data reduction: *SAINT*; program(s) used to solve structure: *SHELXS97* (Sheldrick, 2008 ▶); program(s) used to refine structure: *SHELXL97* (Sheldrick, 2008 ▶); molecular graphics: *ORTEP-3* (Farrugia, 1997 ▶) and *PLATON* (Spek, 2009 ▶); software used to prepare material for publication: *WinGX* (Farrugia, 1999 ▶) and *PLATON*.

## Supplementary Material

Crystal structure: contains datablocks global, I. DOI: 10.1107/S1600536809013841/hb2950sup1.cif

Structure factors: contains datablocks I. DOI: 10.1107/S1600536809013841/hb2950Isup2.hkl

Additional supplementary materials:  crystallographic information; 3D view; checkCIF report

## Figures and Tables

**Table 1:** Hydrogen-bond geometry (Å, °)

*D*—H⋯*A*	*D*—H	H⋯*A*	*D*⋯*A*	*D*—H⋯*A*
N1—H1⋯O4^i^	0.89 (3)	1.89 (3)	2.734 (3)	158 (3)
N3—H3*A*⋯O1	0.83 (3)	2.31 (4)	2.693 (3)	109 (3)
N3—H3*B*⋯O3^ii^	0.86 (3)	2.22 (3)	2.963 (3)	146 (3)
O5—H5*A*⋯O3^i^	0.73 (5)	2.16 (5)	2.871 (3)	166 (7)
O5—H5*B*⋯O2	0.82 (6)	2.07 (6)	2.862 (4)	162 (5)
C4—H4⋯O5^iii^	0.93	2.58	3.423 (5)	152
C9—H9*C*⋯O4^i^	0.96	2.43	3.251 (3)	144
C2—H2⋯*CgC*^i^	0.93	2.84	3.533 (2)	132
S1—O2⋯*CgB*	1.44 (1)	3.17 (1)	3.8430 (9)	107 (1)

Symmetry codes: (i) 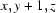; (ii) 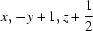; (iii) 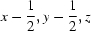. *CgB* and *CgC* are the centroids of the C1/C6/C7/N2/C8/N1 and C10–C15 rings.

**Fig. 1.**
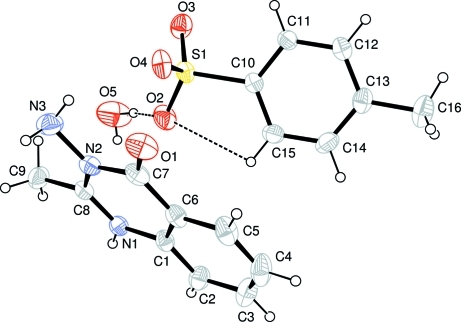
The molecular structure of (I) with displacement ellipsoids drawn at the 30% probability level. H-atoms are shown by small spheres of arbitrary radius and hydrogen bonds by dotted lines.

**Fig. 2.**
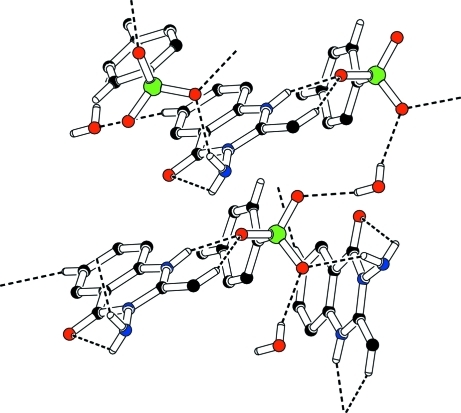
A fragment of the packing of (I) showing R11(5) and R21(6) ring motifs.

## supplementary crystallographic information

### Comment

Cyclic amidines and quinazolinones, are known to possess diverse pharmacological activities as phosphodiesterase inhibitors (Glaser & Traber, 1984), anticonvulsants (Hori *et al.*, 1990), antihypertensives (Glaser & Traber, 1984), vasodilators (Havera, 1979) and fibrinogen receptor antagonists (Liverton *et al.*, 1998).

We now report synthesis and structure of the title compound (I), (Fig. 1), through a reaction of 2-aminobenzoic acid and hydrazine in presence of *p*-toluenesulfonic acid. A one pot, three component, *p*-toluenesulfonic acid catalyzed heterocyclization has yielded colourless prisms of (I) in the form of a *p*-toluenesulfonate salt with one water molecule of crystallization.

The crystal structure of (II) 3-(6-Azabicyclo(3.1.0)hex-2-en-6-yl)-2-((S)-1- hydroxy-2,2-dimethylpropyl)quinazolin-4(3*H*)-one (Atkinson & Meades, 2000) has been published. The title compound has also quinazoline with a chemically different attachements.

In the title compound the two fused rings A (C1—C6) and B(C1/C6/C7/N2/C8/N1) are essentially planar and the ring C (C10—C15) of the *p*-toluenesulfonate anion is of course planar.  The title compound is stabillized due to intra- and intermolecular H-bonding as well as C–H···π and S1==O2···CgB interactions (Table 1). There also exist interactions between the centroids CgA–CgC^i^ [symmetry code: i = *x*, -*y* + 1, *z* + 1/2] and CgB–CgC^i^ at a distance of 3.8648 (12) and 3.9306 (13) Å, respectively. The water molecule connects the *p*-toluenesulfonate ions only. In the title compound there exist *R*_1_^1^(5) and *R*_2_^1^(6) ring motifs (Bernstein *et al.*, 1995), (Fig 2).

### Experimental

A mixture of anthranilic acid (0.14 g, 1 mmol), triethylorthoacetate (0.23 ml, 1.2 mmol), hydrazine hydrate (0.1 ml) and *p*-toluenesulfonic acid (1 g, 5 mmol) was stirred at room temperature for 1 h. After completion of the reaction as indicated by TLC, the reaction mixture was poured into water and allowed to settle, the product precipitated as colourless prisms of (I). The product was filtered, washed with water and dried. m.p. 585–594 K. yield: 72%.

### Refinement

The coordinates of H-atoms connected with water molecule and NH_2_ group were refined. C-bound H-atoms were positioned geometrically (C—H = 0.93–0.96 Å) and refined as riding with *U*_iso_(H) = 1.2*U*_eq_(C, N, O) or 1.5U_eq_(methyl C).

### Crystal data

**Table:**

C_9_H_10_N_3_O^+^·C_7_H_7_O_3_S^−^·H_2_O	*F*(000) = 768
*M**_r_* = 365.40	*D*_x_ = 1.404 Mg m^−^^3^
Monoclinic, *C**c*	Mo *K*α radiation, λ = 0.71073 Å
Hall symbol: C -2yc	Cell parameters from 4390 reflections
*a* = 20.838 (1) Å	θ = 2.9–28.9°
*b* = 6.2769 (3) Å	µ = 0.22 mm^−^^1^
*c* = 14.7897 (7) Å	*T* = 296 K
β = 116.676 (1)°	Prism, colourless
*V* = 1728.56 (14) Å^3^	0.28 × 0.24 × 0.20 mm
*Z* = 4	

### Data collection

**Table:**

Bruker Kappa APEXII CCD diffractometer	4390 independent reflections
Radiation source: fine-focus sealed tube	3952 reflections with *I* > 2σ(*I*)
graphite	*R*_int_ = 0.022
Detector resolution: 7.30 pixels mm^-1^	θ_max_ = 28.9°, θ_min_ = 2.9°
ω scans	*h* = −28→28
Absorption correction: multi-scan (*SADABS*; Bruker, 2005)	*k* = −8→6
*T*_min_ = 0.935, *T*_max_ = 0.958	*l* = −19→20
9546 measured reflections	

### Refinement

**Table:**

Refinement on *F*^2^	Secondary atom site location: difference Fourier map
Least-squares matrix: full	Hydrogen site location: inferred from neighbouring sites
*R*[*F*^2^ > 2σ(*F*^2^)] = 0.034	H atoms treated by a mixture of independent and constrained refinement
*wR*(*F*^2^) = 0.091	*w* = 1/[σ^2^(*F*_o_^2^) + (0.0508*P*)^2^ + 0.3201*P*] where *P* = (*F*_o_^2^ + 2*F*_c_^2^)/3
*S* = 1.00	(Δ/σ)_max_ < 0.001
4390 reflections	Δρ_max_ = 0.19 e Å^−^^3^
243 parameters	Δρ_min_ = −0.22 e Å^−^^3^
2 restraints	Absolute structure: Flack (1983), 2109 Friedal pairs
Primary atom site location: structure-invariant direct methods	Flack parameter: 0.04 (5)

### Special details

**Table:**

Geometry. Bond distances, angles *etc*. have been calculated using the rounded fractional coordinates. All su's are estimated from the variances of the (full) variance-covariance matrix. The cell e.s.d.'s are taken into account in the estimation of distances, angles and torsion angles
Refinement. Refinement of *F*^2^ against ALL reflections. The weighted *R*-factor *wR* and goodness of fit *S* are based on *F*^2^, conventional *R*-factors *R* are based on *F*, with *F* set to zero for negative *F*^2^. The threshold expression of *F*^2^ > σ(*F*^2^) is used only for calculating *R*-factors(gt) *etc*. and is not relevant to the choice of reflections for refinement. *R*-factors based on *F*^2^ are statistically about twice as large as those based on *F*, and *R*- factors based on ALL data will be even larger.

### Fractional atomic coordinates and isotropic or equivalent isotropic displacement parameters (Å^2^)

**Table:**

	*x*	*y*	*z*	*U*_iso_*/*U*_eq_	
O1	0.12729 (10)	0.3093 (3)	0.49282 (14)	0.0676 (6)	
N1	0.15599 (8)	0.8870 (3)	0.40550 (11)	0.0404 (4)	
N2	0.19969 (9)	0.5856 (3)	0.49997 (11)	0.0442 (5)	
N3	0.26093 (12)	0.4718 (4)	0.56765 (18)	0.0611 (7)	
C1	0.08550 (9)	0.8146 (3)	0.36387 (13)	0.0421 (5)	
C2	0.02990 (11)	0.9399 (4)	0.29579 (15)	0.0534 (6)	
C3	−0.03899 (12)	0.8605 (5)	0.25562 (17)	0.0676 (8)	
C4	−0.05286 (12)	0.6617 (5)	0.28300 (18)	0.0723 (9)	
C5	0.00197 (13)	0.5380 (4)	0.34994 (17)	0.0607 (8)	
C6	0.07242 (10)	0.6139 (3)	0.39165 (14)	0.0458 (6)	
C7	0.13149 (12)	0.4861 (3)	0.46325 (15)	0.0483 (7)	
C8	0.21066 (10)	0.7761 (3)	0.46961 (13)	0.0407 (5)	
C9	0.28411 (11)	0.8657 (4)	0.50903 (18)	0.0573 (6)	
S1	0.19585 (2)	0.35554 (7)	0.26006 (3)	0.0413 (1)	
O2	0.19879 (10)	0.5841 (2)	0.27106 (14)	0.0654 (5)	
O3	0.24598 (7)	0.2742 (3)	0.22604 (11)	0.0595 (5)	
O4	0.20275 (8)	0.2499 (3)	0.35157 (10)	0.0551 (4)	
C10	0.10901 (9)	0.2884 (3)	0.16552 (13)	0.0390 (5)	
C11	0.09715 (10)	0.0902 (3)	0.12006 (14)	0.0443 (5)	
C12	0.02813 (11)	0.0315 (3)	0.05242 (15)	0.0504 (6)	
C13	−0.02945 (11)	0.1669 (4)	0.02991 (15)	0.0528 (6)	
C14	−0.01601 (12)	0.3654 (4)	0.07515 (18)	0.0593 (7)	
C15	0.05282 (11)	0.4272 (3)	0.14287 (15)	0.0497 (6)	
C16	−0.10500 (13)	0.1024 (6)	−0.0422 (2)	0.0780 (9)	
O5	0.30414 (16)	0.8511 (4)	0.2540 (3)	0.1157 (13)	
H1	0.1621 (12)	1.020 (4)	0.3909 (17)	0.0485*	
H2	0.03897	1.07432	0.27772	0.0641*	
H3	−0.07674	0.94197	0.20938	0.0809*	
H3A	0.2495 (16)	0.346 (5)	0.551 (2)	0.0734*	
H3B	0.2630 (16)	0.493 (5)	0.626 (2)	0.0734*	
H4	−0.09983	0.61162	0.25573	0.0867*	
H5	−0.00766	0.40379	0.36759	0.0728*	
H9A	0.31436	0.76770	0.49599	0.0688*	
H9B	0.30317	0.88850	0.58061	0.0688*	
H9C	0.28237	0.99873	0.47601	0.0688*	
H11	0.13528	−0.00298	0.13486	0.0532*	
H12	0.02028	−0.10145	0.02150	0.0605*	
H14	−0.05397	0.45945	0.05983	0.0712*	
H15	0.06092	0.56133	0.17269	0.0596*	
H16A	−0.13244	0.08167	−0.00532	0.0934*	
H16B	−0.10387	−0.02800	−0.07543	0.0934*	
H16C	−0.12681	0.21226	−0.09184	0.0934*	
H5A	0.284 (3)	0.949 (8)	0.248 (4)	0.1388*	
H5B	0.271 (3)	0.770 (8)	0.245 (4)	0.1388*	

### Atomic displacement parameters (Å^2^)

**Table:**

	*U*^11^	*U*^22^	*U*^33^	*U*^12^	*U*^13^	*U*^23^
O1	0.0927 (12)	0.0463 (8)	0.0836 (11)	−0.0072 (9)	0.0571 (10)	0.0044 (8)
N1	0.0439 (7)	0.0344 (7)	0.0429 (7)	−0.0061 (6)	0.0195 (6)	−0.0032 (6)
N2	0.0556 (9)	0.0390 (8)	0.0417 (8)	0.0027 (7)	0.0252 (7)	−0.0019 (6)
N3	0.0707 (12)	0.0523 (12)	0.0608 (12)	0.0167 (10)	0.0299 (10)	0.0081 (9)
C1	0.0436 (8)	0.0496 (10)	0.0373 (8)	−0.0099 (7)	0.0218 (7)	−0.0078 (7)
C2	0.0488 (10)	0.0620 (13)	0.0474 (10)	−0.0027 (9)	0.0197 (8)	0.0049 (9)
C3	0.0440 (10)	0.103 (2)	0.0505 (11)	−0.0070 (11)	0.0166 (9)	0.0046 (12)
C4	0.0484 (11)	0.114 (2)	0.0559 (12)	−0.0311 (13)	0.0247 (10)	−0.0118 (13)
C5	0.0637 (12)	0.0704 (15)	0.0578 (12)	−0.0289 (11)	0.0361 (10)	−0.0144 (10)
C6	0.0545 (10)	0.0497 (11)	0.0427 (9)	−0.0131 (8)	0.0304 (8)	−0.0092 (8)
C7	0.0670 (13)	0.0425 (11)	0.0494 (11)	−0.0065 (9)	0.0387 (10)	−0.0048 (8)
C8	0.0475 (9)	0.0374 (9)	0.0388 (8)	−0.0016 (7)	0.0207 (7)	−0.0063 (7)
C9	0.0430 (9)	0.0518 (12)	0.0664 (12)	−0.0025 (9)	0.0150 (9)	−0.0011 (10)
S1	0.0448 (2)	0.0404 (2)	0.0389 (2)	−0.0109 (2)	0.0191 (2)	−0.0002 (2)
O2	0.0721 (9)	0.0420 (7)	0.0751 (10)	−0.0179 (8)	0.0267 (8)	−0.0047 (8)
O3	0.0470 (7)	0.0789 (11)	0.0566 (8)	−0.0051 (7)	0.0269 (7)	−0.0023 (7)
O4	0.0604 (8)	0.0616 (9)	0.0386 (6)	−0.0227 (7)	0.0180 (6)	0.0002 (6)
C10	0.0436 (8)	0.0402 (9)	0.0353 (8)	−0.0043 (7)	0.0197 (7)	0.0000 (7)
C11	0.0449 (9)	0.0429 (10)	0.0463 (9)	−0.0019 (7)	0.0215 (7)	−0.0053 (7)
C12	0.0517 (10)	0.0508 (11)	0.0472 (10)	−0.0104 (9)	0.0209 (8)	−0.0081 (8)
C13	0.0438 (9)	0.0718 (14)	0.0432 (10)	−0.0085 (9)	0.0198 (8)	0.0012 (9)
C14	0.0447 (10)	0.0708 (15)	0.0591 (13)	0.0161 (10)	0.0204 (9)	0.0053 (10)
C15	0.0572 (11)	0.0434 (10)	0.0492 (10)	0.0058 (9)	0.0246 (9)	−0.0023 (8)
C16	0.0473 (12)	0.114 (2)	0.0647 (16)	−0.0166 (13)	0.0180 (11)	−0.0023 (15)
O5	0.1089 (19)	0.0754 (15)	0.205 (3)	−0.0269 (14)	0.108 (2)	−0.0230 (18)

### Geometric parameters (Å, °)

**Table:**

S1—O3	1.4412 (17)	C8—C9	1.482 (3)
S1—O4	1.4551 (16)	C2—H2	0.9300
S1—O2	1.4420 (13)	C3—H3	0.9300
S1—C10	1.7707 (19)	C4—H4	0.9300
O1—C7	1.210 (3)	C5—H5	0.9300
O5—H5B	0.82 (6)	C9—H9C	0.9600
O5—H5A	0.73 (5)	C9—H9B	0.9600
N1—C1	1.390 (3)	C9—H9A	0.9600
N1—C8	1.308 (3)	C10—C11	1.383 (3)
N2—N3	1.412 (3)	C10—C15	1.375 (3)
N2—C7	1.418 (3)	C11—C12	1.383 (3)
N2—C8	1.333 (3)	C12—C13	1.384 (3)
N1—H1	0.89 (3)	C13—C14	1.382 (3)
N3—H3A	0.83 (3)	C13—C16	1.507 (4)
N3—H3B	0.86 (3)	C14—C15	1.386 (3)
C1—C6	1.390 (3)	C11—H11	0.9300
C1—C2	1.388 (3)	C12—H12	0.9300
C2—C3	1.377 (4)	C14—H14	0.9300
C3—C4	1.382 (4)	C15—H15	0.9300
C4—C5	1.369 (4)	C16—H16C	0.9600
C5—C6	1.396 (4)	C16—H16A	0.9600
C6—C7	1.453 (3)	C16—H16B	0.9600
			
O2—S1—C10	107.61 (11)	C2—C3—H3	119.00
O2—S1—O3	113.02 (12)	C5—C4—H4	120.00
O2—S1—O4	111.53 (11)	C3—C4—H4	120.00
O4—S1—C10	105.38 (9)	C4—C5—H5	120.00
O3—S1—O4	112.26 (10)	C6—C5—H5	120.00
O3—S1—C10	106.50 (9)	C8—C9—H9A	109.00
H5A—O5—H5B	96 (7)	C8—C9—H9B	109.00
C1—N1—C8	123.39 (18)	C8—C9—H9C	109.00
N3—N2—C7	118.69 (19)	H9A—C9—H9B	109.00
N3—N2—C8	117.1 (2)	H9A—C9—H9C	109.00
C7—N2—C8	124.06 (17)	H9B—C9—H9C	110.00
C1—N1—H1	116.1 (17)	S1—C10—C11	119.63 (15)
C8—N1—H1	120.3 (17)	S1—C10—C15	119.88 (15)
N2—N3—H3A	103 (2)	C11—C10—C15	120.33 (18)
H3A—N3—H3B	109 (3)	C10—C11—C12	119.49 (19)
N2—N3—H3B	105 (2)	C11—C12—C13	121.21 (19)
C2—C1—C6	121.2 (2)	C12—C13—C14	118.2 (2)
N1—C1—C2	120.53 (19)	C14—C13—C16	120.3 (2)
N1—C1—C6	118.30 (17)	C12—C13—C16	121.5 (2)
C1—C2—C3	118.5 (2)	C13—C14—C15	121.4 (2)
C2—C3—C4	121.0 (2)	C10—C15—C14	119.38 (19)
C3—C4—C5	120.5 (3)	C12—C11—H11	120.00
C4—C5—C6	119.8 (2)	C10—C11—H11	120.00
C5—C6—C7	120.83 (19)	C11—C12—H12	119.00
C1—C6—C7	120.12 (19)	C13—C12—H12	119.00
C1—C6—C5	119.05 (19)	C15—C14—H14	119.00
N2—C7—C6	114.30 (17)	C13—C14—H14	119.00
O1—C7—N2	119.2 (2)	C10—C15—H15	120.00
O1—C7—C6	126.5 (2)	C14—C15—H15	120.00
N2—C8—C9	120.72 (19)	C13—C16—H16B	109.00
N1—C8—N2	119.8 (2)	C13—C16—H16C	109.00
N1—C8—C9	119.51 (19)	C13—C16—H16A	109.00
C1—C2—H2	121.00	H16A—C16—H16C	109.00
C3—C2—H2	121.00	H16B—C16—H16C	109.00
C4—C3—H3	120.00	H16A—C16—H16B	109.00
			
O4—S1—C10—C11	−78.48 (18)	N1—C1—C6—C7	0.6 (3)
O4—S1—C10—C15	96.94 (18)	C2—C1—C6—C5	0.1 (3)
O2—S1—C10—C11	162.40 (17)	C1—C2—C3—C4	0.7 (4)
O2—S1—C10—C15	−22.2 (2)	C2—C3—C4—C5	−0.8 (4)
O3—S1—C10—C11	40.94 (19)	C3—C4—C5—C6	0.6 (4)
O3—S1—C10—C15	−143.64 (17)	C4—C5—C6—C7	179.7 (2)
C1—N1—C8—C9	179.25 (18)	C4—C5—C6—C1	−0.2 (3)
C8—N1—C1—C6	−0.1 (3)	C5—C6—C7—N2	−179.2 (2)
C1—N1—C8—N2	−1.8 (3)	C1—C6—C7—O1	−179.4 (2)
C8—N1—C1—C2	−179.68 (19)	C1—C6—C7—N2	0.7 (3)
N3—N2—C7—O1	2.0 (3)	C5—C6—C7—O1	0.7 (4)
C7—N2—C8—C9	−177.77 (19)	S1—C10—C11—C12	174.65 (16)
N3—N2—C7—C6	−178.09 (19)	C15—C10—C11—C12	−0.8 (3)
C8—N2—C7—O1	177.4 (2)	S1—C10—C15—C14	−174.44 (17)
C8—N2—C7—C6	−2.7 (3)	C11—C10—C15—C14	0.9 (3)
N3—N2—C8—N1	178.78 (19)	C10—C11—C12—C13	−0.5 (3)
N3—N2—C8—C9	−2.3 (3)	C11—C12—C13—C14	1.5 (3)
C7—N2—C8—N1	3.3 (3)	C11—C12—C13—C16	−178.8 (2)
C2—C1—C6—C7	−179.83 (19)	C12—C13—C14—C15	−1.3 (4)
N1—C1—C6—C5	−179.51 (19)	C16—C13—C14—C15	179.0 (2)
N1—C1—C2—C3	179.3 (2)	C13—C14—C15—C10	0.1 (3)
C6—C1—C2—C3	−0.3 (3)		

### Hydrogen-bond geometry (Å, °)

**Table:**

*D*—H···*A*	*D*—H	H···*A*	*D*···*A*	*D*—H···*A*
N1—H1···O4^i^	0.89 (3)	1.89 (3)	2.734 (3)	158 (3)
N3—H3A···O1	0.83 (3)	2.31 (4)	2.693 (3)	109 (3)
N3—H3B···O3^ii^	0.86 (3)	2.22 (3)	2.963 (3)	146 (3)
O5—H5A···O3^i^	0.73 (5)	2.16 (5)	2.871 (3)	166 (7)
O5—H5B···O2	0.82 (6)	2.07 (6)	2.862 (4)	162 (5)
C4—H4···O5^iii^	0.93	2.58	3.423 (5)	152
C9—H9C···O4^i^	0.96	2.43	3.251 (3)	144
C2—H2···CgC^i^	0.93	2.84	3.533 (2)	132
S1—O2···CgB	1.4420 (13)	3.169 (2)	3.8430 (9)	106.86 (10)

Symmetry codes: (i) *x*, *y*+1, *z*; (ii) *x*, −*y*+1, *z*+1/2; (iii) *x*−1/2, *y*−1/2, *z*.
